# Rapidly starting antiretroviral therapy to improve outcomes among disadvantaged groups

**DOI:** 10.1097/QAD.0000000000003052

**Published:** 2021-10-01

**Authors:** Nathan Ford, Keith W. Crawford, Wole Ameyan

**Affiliations:** a Global HIV, Hepatitis and Sexually Transmitted Infections Programme, World Health Organization, Geneva, Switzerland; b Centre for Infectious Disease Epidemiology and Research, School of Public Health and Family Medicine, University of Cape Town, South Africa; c Therapeutic Research Program, Division of AIDS, National Institute of Allergy and Infectious Disease, National Institutes of Health, Rockville, MD, USA.

**Keywords:** antiretroviral therapy, HIV, men, West Africa

Fifteen years ago, a review of HIV programmes across southern Africa concluded that fewer men were receiving antiretroviral therapy (ART) compared with women [1]. Recognition of this delayed engagement with care, which persists today [2], has grown in recent years, with several recent studies showing that, compared with women, men present with more advanced HIV disease and associated higher mortality risk, are less likely to start ART and are less likely to be retained in care [3,4].

In this issue of *AIDS*, a prospective cohort study [5] provides outcomes of rapid ART initiation among MSM from four countries in West Africa (Burkina Faso, Côte d’Ivoire, Mali, and Togo) between 2015 and 2019. This study is important as it reflects several key challenges in the HIV response. In addition to the challenge of increasing access to ART among men in general, there is a need for more evidence to guide interventions to improve uptake and outcomes of ART for MSM in low-income and middle-income settings [6]. ART coverage and viral suppression among MSM in Africa remain much lower than required to achieve global targets [7]. In addition, as highlighted by an Médecins Sans Frontières report in 2016 [8], and echoed by UNAIDS [9], improvements in access to HIV treatment and care has been slower in West Africa compared with other regions. HIV-positive MSM in West Africa, therefore, represent a distinctly disadvantaged group.

Rapid initiation of ART is one way to increase the number of people starting treatment by reducing losses between testing and treatment. Since 2017, the World Health Organization (WHO) recommends starting ART within 7 days of an HIV diagnosis, and suggests the possibility of starting treatment on the same day of diagnosis [10]. In 2021, WHO also recommended that ART can be started outside of a health facility [11], opening the way for same day ART start as part of community-testing approaches [12]. These guidelines also put forward a good practice statement aimed at promoting uptake of same day ART start, promoting approaches to improve uptake, treatment adherence and retention such as tailored patient education, counselling and support.

The study from West Africa clearly demonstrated the benefits of this approach for MSM. Two-thirds of men (65%) who were offered treatment started within 7 days, and a quarter (24%) on the same day, reflecting a high level of acceptability among a relatively healthy population (96% were WHO Clinical Stage I and median CD4^+^ cell count was 398 cells/μl). In multivariate analysis, rapid initiation improved viral suppression [adjusted odds ratio (aOR) 6.96, 95% confidence interval (CI) 1.98–24.46], and there was a tendency towards improved viral suppression associated with same-day ART start in univariate analysis (OR 3.33, 95% CI 0.90–12.31) [13]. Reassuringly, rapid ART initiation and same day ART start were not associated with increased disengagement from care, a concern that has been reported by several observational studies [14].

Overall, however, a third of those starting treatment were not retained in care after 3 years of follow-up, with the majority of people disengaging within the first 12 months after start of treatment. This effect of reduced retention-in-care must be well understood to determine the value of this intervention among African MSM. Is it a negative consequence of rapid ART initiation or is it a feature of this particular demographic? Some studies have observed an increased risk of loss to care among pregnant women following rapid ART initiation, underscoring the importance of providing postinitiation follow-up and support for populations at risk of disengagement from care [14]. Other studies have reported that African MSM have worse outcomes across a number of important indicators, including increased loss to follow-up [14]. A study among HIV-positive MSM in Kenya found that, in spite of employing evidence-based supportive measures, they experienced lower virologic suppression rates compared with adults in other ART programs in Africa (including the general adult population in Kenya) [15]. Men who acquired coping self-efficacy skills, in this environment of intensive structural stigma, had better outcomes, including engagement in the health system. In a study of rapid ART initiation among men in the United States, there were no differences in loss to care comparing those receiving the intervention and those receiving standard care [16].

In addition to recommending support for retention in care, the latest WHO guidelines recommends that HIV programmes include activities to trace people who have disengaged from care and provide support for re-engagement [12]. Individuals who have simply relocated and continued care at another facility may be misclassified as being lost to care [16]. The benefits of rapid ART initiation will only be realized if people who start treatment are provided with appropriate support to remain in care over the long-term, and nonjudgementally supported to reengage in care if they need to interrupt care for whatever reason.

The impact of the additional burden of the stigma of being a gay male in sub-Saharan Africa must not be underestimated. Homosexuality is criminalized in many countries in sub-Saharan Africa with penalties as severe as imprisonment and death. Beyond the extreme violation of human rights brought on by these laws, they have an adverse effect on access to care and well being of HIV-positive MSM. A recent systematic review found that African countries with the most severe antigay legislation had substantially reduced rates of HIV testing among MSM compared with countries with less severe legislation (e.g. testing in the last 12 months, 35.5 vs. 49.3%, *P* = 0.01) [7].

The types of co-infections that may be present at the time of ART initiation can determine which antiretrovirals are used, the dosage, as well as the timing for starting ART. Currently, most people are started on a dolutegravir-based first-line regimen; if rifampicin is also being administered for tuberculosis (TB) treatment, the dosage should be raised to 50 mg twice daily [12]. Unlike efavirenz, dolutegravir is active against HIV-2, which is common in West Africa. The key contra-indication for rapid ART start is when cryptococcal meningitis is diagnosed; in such individuals, ART initiation should be deferred 4–6 weeks from the initiation of antifungal treatment [17] People with advanced HIV disease should be given priority for initiating ART as they are at higher risk of death. They should be evaluated for the risk or presence of TB and cryptococcal meningitis for which point-of-care diagnostic tests exist [12].

Individuals with pretreatment drug resistance, which may occur in greater than 10% of individuals starting ART in some countries in sub-Saharan Africa [18], are more likely to experience virologic failure. This again favours initiating with dolutegravir-containing regimens in resource-limited settings where baseline drug resistance testing is currently not recommended prior to starting ART. Point-of-care genotypic resistance tests may make resistance testing more widely available but the role of such tests in a public health approach to ART delivery remains to be determined [19].

In conclusion, rapid ART initiation is operationally feasible in African MSM and may have an important role in controlling the epidemic. Additional resources are needed to ensure sustainable benefit for this vulnerable group.

## Acknowledgements

### Conflicts of interest

There are no conflicts of interest.
